# Artificial intelligence-powered copilots for precision diagnosis and surgical assessment of histological growth patterns in resectable colorectal liver metastases: a prospective study

**DOI:** 10.1097/JS9.0000000000002922

**Published:** 2025-07-04

**Authors:** Ruichong Lin, Yongjian Chen, Yanchun Li, Yujie Tan, Chao Wang, Zehua Wang, Mengyang Sun, Lin Wang, Yufei Wu, Qiyun Ou, Lui Ng, Xiaoxi Zhang, Weidong Pan, Zongyan Li, Zuxiao Chen, Zheyu Zheng, Xiaoming Huang, Lei Zhang, Jingsong Sun, Zaopeng He, Nannan Li, Yunfang Yu, Dawei Zhang

**Affiliations:** a Faculty of Innovation Engineering, School of Computer Science and Engineering, Institute for AI in Medicine, Faculty of Medicine, Faculty of Humanities and Arts, Macau University of Science and Technology, Taipa, Macao, China; b Department of Computer and Information Engineering, Guangzhou Huali College, Guangzhou, China; c Guangzhou National Laboratory, Guangzhou, China; d Foshan Shunde Lecong Hospital, Foshan, China; e Department of Medicine Solna, Center for Molecular Medicine, Karolinska Institutet, Stockholm, Sweden; f UmedEVO and UmedREVO Artificial Intelligence Technology (Guangzhou) Co., Ltd, Guangzhou, China; g Guangdong Provincial Key Laboratory of Malignant Tumor Epigenetics and Gene Regulation, Guangdong-Hong Kong Joint Laboratory for RNA Medicine, Department of Medical Oncology, Department of Pathology, Sun Yat-sen Memorial Hospital, Sun Yat-sen University, Guangzhou, China; h Department of Pancreatic Hepatobiliary Surgery, Department of Pathology, Department of Breast Surgery, Biomedical Innovation Center, The Sixth Affiliated Hospital, Sun Yat-Sen University, Guangzhou, China; j Department of Surgery, School of Clinical Medicine, Li Ka Shing Faculty of Medicine, The University of Hong Kong, Hong Kong, China; k School of Computer Science and Engineering & Key Laboratory of Machine Intelligence and Advanced Computing, Sun Yat-sen University, Guangzhou, China; l Guangdong Provincial Key Laboratory of Cancer Pathogenesis and Precision Diagnosis and Treatment, Joint Big Data Laboratory, Department of Medical Oncology, Shenshan Medical Center, Memorial Hospital of Sun Yat-sen University, Shanwei, China; m Department of Breast Surgery, The First Affiliated Hospital, Jinan University, Guangzhou, China

**Keywords:** artificial intelligence in diagnosis, colorectal liver metastasis, desmoplastic classification, histopathological growth patterns, vision transformer

## Abstract

**Background:**

Colorectal cancer (CRC) is a leading cause of mortality in China, with metastasis significantly contributing to poor outcomes. Histopathological growth patterns (HGPs) in colorectal liver metastasis (CRLM) provide vital prognostic insights, yet the limited number of pathologists highlights the need for auxiliary diagnostic tools. Recent advancements in artificial intelligence (AI) have demonstrated potential in enhancing diagnostic precision, prompting the development of specialized AI models like COFFEE to improve the classification and management of HGPs in CRLM patients.

**Methods:**

This study developed a Transformer-based deep learning model, COFFEE, for the precise classification of colorectal cancer subtypes using whole-slide images (WSIs) from 431 patients diagnosed with colorectal cancer liver metastasis. The model was pretrained using DINO on 1442 WSIs from the TCGA-COAD cohort, utilizing a Vision Transformer (ViT) architecture to extract 384-dimensional feature vectors from 256 × 256 pixel patches. The proposed model integrates a Transformer-based Multiple Instance Learning (TransMIL) framework, which effectively aggregates spatial and morphological information through multi-head self-attention and Pyramid Position Encoding Generator (PPEG) modules. This design enables efficient handling of large instance sequences within WSIs, allowing for accurate binary and four-class classification. The model was validated on 972 WSIs from a recent dataset, demonstrating its robustness and clinical applicability. After testing the model with internal and prospective cohorts, a direct comparison between conventional and AI-assisted pathology assessment was also performed.

**Results:**

A total of 431 patients were included in three cohorts: training (*n* = 297), testing (*n* = 104), and prospective (*n* = 30). Desmoplastic tumors were associated with longer overall survival (OS, 53.6 vs. 31.9 months, *P* = 0.002) and progression-free survival (PFS, 25.2 vs. 10.7 months, *P* < 0.001) compared to non-desmoplastic tumors. The COFFEE binary classification model achieved high predictive performance with area under the ROC curve (AUC) values of 0.961 in the training, 0.935 in the testing, and 1.000 in the prospective cohort. The four-class model also showed strong performance, with AUCs of 0.961 and 0.966 in the training and testing cohorts, and 0.985 in the prospective cohort. AI-assisted models helped junior pathologists achieve an accuracy of 94.7% (vs. 85.9%) and reduced diagnostic time by 36%, improving both accuracy and speed.

**Conclusion:**

This study developed an AI model for HGP classification in colorectal cancer liver metastasis, achieving high accuracy in both binary classification and four-class classification models. The model demonstrated potential for improving diagnostic precision and guiding post-surgery treatment strategies, with AI-assisted pathologists surpassing traditional methods in a prospective cohort.

## Introduction

Colorectal cancer (CRC) is the second most prevalent malignancy in China, with metastasis being the primary cause of CRC-related mortality and a significant challenge following curative treatment^[1]^. Approximately 50–60% of CRC patients eventually develop colorectal liver metastases (CRLM), with 80–90% of these cases deemed unresectable. Nonetheless, selected patients who undergo resection of liver metastases have demonstrated promising outcomes, with improved progression-free survival (PFS) and overall survival (OS)^[2]^. These findings highlight the urgent need for accurate diagnostic models for CRLM.

Histopathological growth patterns (HGPs) in CRLM, representing the interactive boundaries between tumor margins and adjacent liver parenchyma, offer critical insights into tumor biology^[3]^. These patterns, including angiogenesis, apoptosis, and immune responses, are key prognostic factors in CRC^[4]^. In 2017, an international consensus established three HGP classifications: desmoplastic HGP (dHGP), replacement HGP (rHGP), and pushing HGP (pHGP)^[5]^. The clinical relevance of these HGPs has been well-documented, with studies showing significantly better OS and PFS in patients with desmoplastic HGP compared to those with non-desmoplastic HGP, who also derive greater benefit from adjuvant systemic chemotherapy^[6]^. Additionally, the preparation and assessment of HGPs using hematoxylin and eosin (H&E)-stained tissue sections are straightforward, reproducible, and reliable for prognostic evaluation. Given the critical role of HGP classification in guiding treatment decisions for CRLM patients, accurate diagnostic tools are essential^[7]^. However, with only 20 400 registered pathologists in China as of 2022, there is a pressing need for auxiliary diagnostic tools to support CRC management^[8]^.

Recent advances in artificial intelligence (AI) have significantly impacted oncology, particularly in cancer diagnosis, prognosis, and treatment selection, by integrating omics and histopathological data across various cancer types, including lung, breast, and CRC^[9]^. For instance, AI techniques such as optimal policy trees (OPTs) have been employed to determine optimal resection margin widths for CRLM patients, revealing that a 7 mm margin correlates with the longest survival in KRAS-mutated CRLM patients^[10]^. Moreover, AI models have been used to analyze texture features in T2-weighted MR images, accurately predicting pathological complete response in locally advanced rectal cancer patients post-neoadjuvant chemoradiotherapy^[11]^. Another study introduced a visual-language foundation model for computational pathology, achieving state-of-the-art performance in tasks like image classification and segmentation^[12]^. Despite these advancements, there remains a significant gap in AI applications specifically for HGP classification in CRLM. Addressing this gap is crucial for developing specialized AI models that can enhance diagnostic precision and ultimately improve patient outcomes.

**HIGHLIGHTS**

- Developed COFFEE, a Transformer model to classify HGPs in CRLM from WSIs.
- COFFEE achieved AUCs of 0.961 train, 0.935 test, 1.000 prospective in HGP tasks.
- AI boosted pathologist accuracy from 85.9% to 94.7%, cut time by 36% in diagnosis.
- COFFEE enables accurate HGP classification to guide prognosis and treatment in CRLM.

In this study, we developed and validated a deep learning model, COFFEE, designed for the precise classification of HGPs in CRLM patients. The model utilizes WSIs from surgical specimens of CRLM patients as input features to predict both binary and four-class HGP classifications. COFFEE is developed based on the DINO self-distillation framework and utilizes a Vision Transformer (ViT) architecture. The model was initially pretrained on the TCGA-Colon Adenocarcinoma dataset in order to learn robust and generalizable histopathological features. The performance of COFFEE was prospectively evaluated and compared against that of experienced pathologists, aiming to assess its diagnostic utility in real-world clinical scenarios. The results suggest that COFFEE improves diagnostic accuracy and supports clinical decision-making in the management of colorectal liver metastases. These findings highlight its potential as a valuable adjunct in clinical workflows to enhance patient outcomes.

## Methods

### Study design and patients

**Figure 1.**
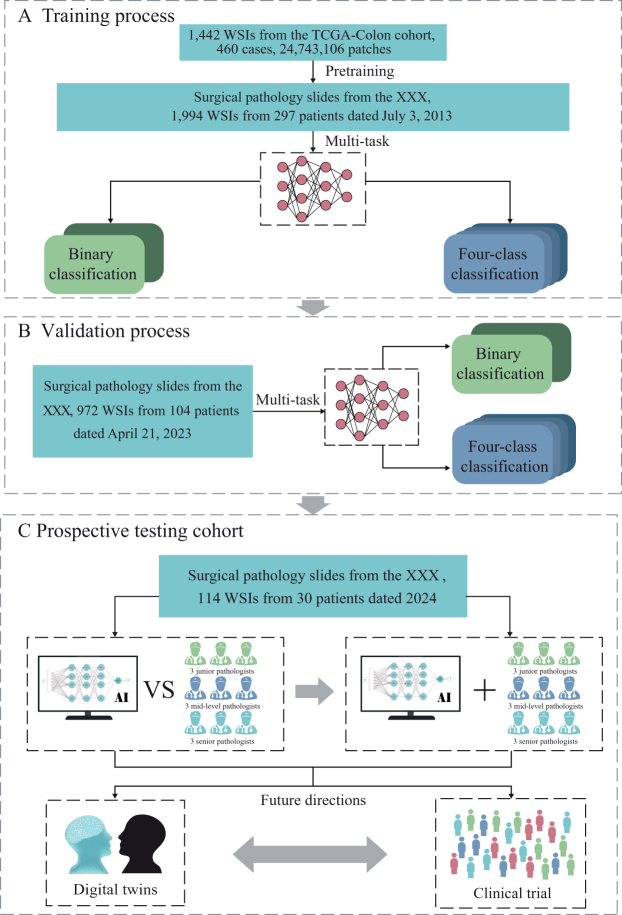
Application of the COFFEE model in clinical classification of CRLM. (A) Training process: The model was pretrained using the TCGA-Colon cohort, followed by further training with CRLM pathology slides from SAHSYSU (2013). The model demonstrated high accuracy and speed in binary and four-class HGP classifications, aiding pathologists with rapid diagnostic results; (B) Testing process: The COFFEE model was tested using 2023 CRLM pathology slides from SAHSYSU. Results from data collected a decade earlier confirmed the model’s reliability in clinical practice; (C) prospective validation cohort: In 2024, pathology slides from 30 CRLM patients were used to evaluate the COFFEE model. The left panel compared the model’s performance with that of junior, intermediate, and senior pathologists in binary and four-class classifications. The right panel assessed the impact of COFFEE model assistance on pathologist performance. The results showed that the COFFEE model achieved comparable accuracy to senior pathologists with faster classification speeds, significantly enhancing the accuracy and speed of pathologists in WSI-based CRLM classification. The model also has potential for future applications in digital twin technology and clinical trials. SAHSYSU, the Sixth Affiliated Hospital of Sun Yat-sen University; HGP, histopathological growth pattern; WSI, whole slide images; CRLM, colorectal liver metastasis.

This study retrospectively collected pathological samples of liver metastases from 431 patients with colorectal cancer who underwent surgical resection at the Sixth Affiliated Hospital of Sun Yat-sen University (SAHSYSU). The training dataset comprised 1994 whole-slide images (WSIs) from 297 patients, originating from an earlier batch dated 3 July 2013, and the testing dataset included a more recent collection of 972 WSIs from 104 patients, dated 21 April 2023 (Fig. 1). In 2024, two prospective experiments were conducted using WSIs obtained from liver metastases resected during surgeries performed in the same year. One prospective experiment involved a human–AI competition, where nine pathologists and AI independently interpreted the same set of WSIs to determine binary and quaternary classifications. This experiment aimed to assess the performance of the model. Another prospective experiment evaluated the effectiveness of AI-assisted classification, where an additional group of nine pathologists performed the same classification tasks with AI support, further validating the model’s clinical applicability. Both prospective experiments utilized the same set of 114 WSIs derived from 30 patients, which were assessed in two separate experiments involving two distinct groups of pathologists (each group consisting of nine independent board-certified pathologists). While the WSI order and content remained consistent across both phases to ensure comparability, the two reader groups were independent and blinded to each other’s results.

The inclusion criteria were as follows: (1) maximum diameter of resected metastatic lesions ≥2 cm, (2) sufficient specimen tissue at the tumor–liver tissue interface on HE slides for evaluating histological growth patterns, and (3) availability of pathology slides along with baseline clinical, biological, and pathological features. The exclusion criteria were: (1) tissue sections from biopsy specimens, (2) absence of viable tumor tissue in metastatic lesions, and (3) lesions previously treated with ablation followed by surgical resection, resulting in inadequate tissue slide quality.

In this study, the truth label of HGP classification was assessed by an expert cytopathologist from the Pathology Department of the SAHSYSU. The BioStrong SQS-600P scanner was used in this project, with images initially captured in the sdpc format and subsequently converted to the svs format for analysis. The work has been reported in line with the STROCSS 2025 criteria (www.strocssguideline.com), and this study’s design, conduct, and reporting adhere to established guidelines^[13]^. This study was registered at ClinicalTrials.gov (NCT06936098):

### Improvement and preprocessing of pathology image quality

The data processing pipeline begins with WSIs as input to ensure high-resolution and consistent feature extraction. To ensure accuracy, all WSIs were scanned at a 20 × magnification, excluding slides with lower resolutions. This study gathered 2966 hematoxylin and eosin (HE)-stained WSIs from a cohort of 424 patients, including cohorts from the Pathology Department of SAHSYSU. This study deployed an automated WSI layout tailored to each image size, leveraging the Clustering-constrained Attention Multiple Instance Learning (CLAM) method^[14]^. After outlining the graphical boundaries and removing background using morphological features, each WSI was downscaled into 256 × 256 pixel patches, with a magnification factor of 20 × . Feature extraction utilizes a pretrained Vision Transformer (ViT) model on TCIA pan-cancer WSIs data, producing 384-dimensional feature vectors per patch^[15]^.

### Details of model pretraining

This study employs Distillation with NO labels (DINO) for pretraining on 1442 WSIs from the TCGA-COAD (Colon Cancer) cohort. Features are extracted from each WSI, which is uniformly divided into 256 × 256 patches, resulting in a total of 24 743 106 patches^[16]^. The model encoder applies the ViT-Small architecture, and the input patch level is 16 × 16, and the batch size is set to the 64. The ViT small architecture comprises 12 encoder layers^[17]^, incorporating a multi-head self-attention mechanism and an MLP, facilitating global interactions between fragments and capturing long-range dependencies in the images. The base learning rate was set to 5 $\times 10^{-4}$, and the minimum learning rate is $1\times 10^{-6}$ with 10 epochs to warm up from total 100 epochs. We also freeze the final layer of the DINO head and normalize it. The slide encoder pretraining utilized a 80 GB A100 GPUs and was completed in approximately 2 days.

### Development and validation of COFFEE

In this study, we developed a deep learning model for **co**lorectal cancer subtype classi**f**ication based on a Trans**f**ormer archit**e**ctur**e** (COFFEE), using surgical pathological images from patients^[18]^. The architecture of the network comprises two key stages: building pretrained models and feature representation learning from postoperative WSIs. Additionally, this deep learning framework includes crucial stages such as quality control and classification. By utilizing this cutting-edge deep learning model, this study aims to establish a system that provides valuable insights for predicting colorectal cancer subtypes.

Through DINO-based knowledge distillation, the model learns data-efficient and interpretable features in histology images, with different attention heads capturing distinct morphological phenotypes^[19]^. Following that, for feature representation learning, a multilayer perceptron (MLP) with two fully connected (FC) layers is utilized to map the hidden states to a latent space. Subsequently, a self-attention module is employed to dynamically adjust the importance of these features for specific tasks. This module comprises two FC layers with tanh and sigmoid activation functions, with the final FC layer computing the element-wise product of these two activations. Attention scores are computed using the softmax function.

**Figure 2.**
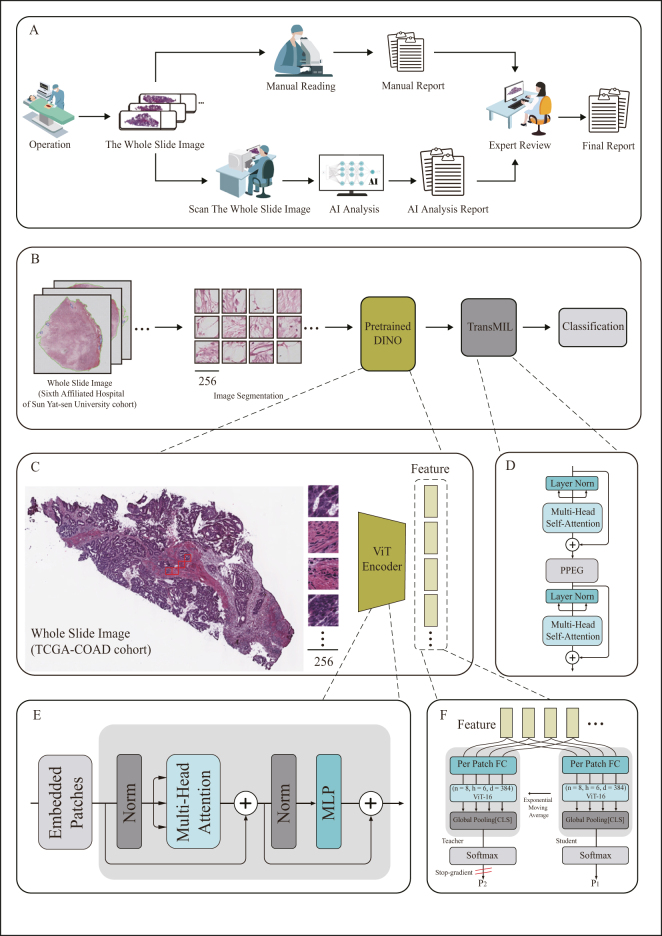
The workflow of the study. (A) Histopathological classification: Histopathological samples from liver cancer patients were digitized and classified at the WSI level using the COFFEE model, to assist pathologists. The final diagnosis was reviewed by experienced pathologists; (B) COFFEE model architecture: Each WSI was segmented into 256 × 256 pixel patches, which were processed by the pre-trained DINO model for feature extraction, followed by classification using the TransMIL framework for binary and four-class CRLM classification; (C) Pretraining framework: The TCGA-Colon cohort was used for pretraining. WSIs were segmented into patches, which were passed through a ViT-Encoder for feature extraction; (D) ViT encoder framework: Images were divided into 256 × 256 patches, linearly embedded, and positional embeddings were added before processing through the Transformer encoder; (E) TransMIL framework: TransMIL employs multi-head self-attention, layer normalization, and residual connections to model region relationships and improve gradient flow, optimizing pathology image processing; (F) DINO framework: DINO uses knowledge distillation for self-supervised learning, with the student network trained to match the teacher network’s probability distribution, enhanced by local and global pooling. WSI, whole slide images; DINO, Distillation with NO labels; CRLM, colorectal liver metastasis; ViT, Vision Transformer; TransMIL, Transformer-based Multiple Instance Learning.

Following the automated feature extraction from WSI patches, we adapt a pathological deep learning model tailored for precise classification of colorectal cancer into distinct subtypes. This model leverages a Transformer-based WSI classification method^[20]^, that comprehensively considers the correlations among different instances (patches) within the same bag (WSI). In this approach, the function h encodes spatial relationships among instances, while the Pooling Matrix P employs self-attention mechanisms for information aggregation. Given a set of bags { $X_{1}$, $X_{2}$,…, $X_{b}$}, where each bag $X_{i}$ contains multiple instances { $x_{i,1},\;x_{i,2},\ldots \;,x_{i,n}$} and a corresponding label $Y_{i}$, the objective is to learn the mappings: X→T→Y. Here, X represents the bag space, T represents the Transformer space, and Y represents the label space. Additionally, the architecture includes a Token Pyramid Transformer (TPT) module comprising two Transformer layers and a position encoding layer. The Transformer layers are designed to aggregate morphological information, while the Pyramid Position Encoding Generator (PPEG) encodes spatial information. An overview of the proposed Transformer-based Multiple Instance Learning (TransMIL) framework is illustrated in Figure 2.

Specifically, the formula can be shown as:



Where MSA denotes Multi-head Self Attention, LN denotes the Layer normalization, MLP denotes Multi Layer Percenptor, and the self attention would repeat l times. To deal with the long instances sequence problem in WSIs, the softmax in TPT adopts the Nystrom Method proposed in Wang et al. ^[21]^. The approximated self-attention form Ŝ can be defined as:



Where $\overset{ˉ}{Q}$ and $\overset{ˉ}{K}$ are the m selected landmarks from the original n dimensional sequence of Q and K, and $({)}^{+}$ means a Moore–Penrose pseudoinverse of it. In reference to the low-rank decomposition, we use the values m of K and Q at low latitude, respectively, to reduce the complexity of the computation from O ( $n^{2}$) to O(n). By doing this, the TPT module with approximation processing can satisfy the case where a bag contains thousands of tokens as input.

### Model construction

During pretraining, we used DINO with ViT Small as the backbone model. To speed up training, we divided the input pixels into 16 × 16 patches and employed mixed precision. We maintained the default DINO output head dimension of 65 536 to match the high dimensionality of our extracted patch features. To maintain training stability, we applied normalization in the final layer. The momentum for updating the teacher network was set to 0.996, and we did not use batch normalization in the projection head. For optimization, we trained with fp16 precision, starting weight decay at 0.04 and ending at 0.4, with a batch size of 64, warmup epochs set to 10, a learning rate of 5 $\times 10^{-4}$, and used the AdamW optimizer.

For both binary classification and four-class classification, we set the same hyperparameters and configured them in a single configuration file. Specifically, during the model training phase, we used the cross-entropy loss function and the Adam optimizer, initialized all the model parameters, and set the learning rate to 5 $\times 10^{-4}$. For both binary and four-class classification, we set the training to run for 100 epochs with a batch size of 1 and implemented an early stopping mechanism with a patience of 20 epochs. All other parameters were kept at their default values. The model was built and run using a single A100 GPU with 80 GB of memory, and the entire process took one day to complete.

To robustly validate the clinical efficacy of COFFEE, we employed a targeted strategy to assess its real-world performance. During the validation phase, the parameters of the pretrained binary and four-class models were frozen to prevent any updates, ensuring that the features learned by the model remained unchanged. This approach guaranteed the stability of the model’s performance and provided a reliable evaluation of its capability in a clinical setting.

### Visualization and interpretation of the model

To accurately analyze the image features and corresponding regions in WSIs influencing the model’s output, this study integrates advanced visualization techniques with the CLAM framework to enhance model interpretability. Initially, attention mechanisms are applied to extract patches from WSIs, and attention scores are generated for each region. These scores are linearly transformed using a softmax function and visualized as heatmaps. The attention mechanism highlights regions with high attention scores as potential diagnostic tumor tissue, while areas with lower scores are classified as normal tissue. To further refine this process, the original TransMIL algorithm’s attention mechanism is used to compute attention scores, with 0-value padding removed to ensure accurate visualization. The CLAM framework simplifies the prediction process by eliminating the need for WSI annotations, enhancing the efficiency of identifying significant regions. The process begins by extracting the foreground of each pathological tissue, followed by segmenting each foreground into smaller regions, where each segment’s features are extracted using the pre-trained model. The final attention scores are computed via linear attention mechanism computation, resulting in the visualization of heatmaps for each classification of colorectal cancer liver metastasis, including all four HGP types and the binary classification. This visualization strategy enhances model interpretability by linking morphological regions to prediction scores, thereby offering valuable insights for clinical decision-making.

### Statistical analyses

Chi-squared tests were used to compare categorical variables, while the Mann–Whitney U-test or Kruskal–Wallis H-test was applied to continuous variables. Median survival between groups was assessed using the log-rank test. Model performance was evaluated based on the AUC, accuracy, sensitivity, specificity, positive predictive value (PPV), and negative predictive value (NPV), calculated using the “pROC” and “epiR” packages in R software. A *P-*value < 0.05 was considered statistically significant. All statistical analyses were performed using R software (version 4.3.3).

## Results

### Patient characteristics

The data were derived from pathological records of colorectal cancer liver metastasis patients at SAHSYSU, including a training cohort (*n* = 297), a testing cohort (*n* = 104), and a prospective cohort (*n* = 30), with 1994, 972, and 114 WSIs, respectively. The median follow-up durations were 23 months (IQR: 16–38), 11 months (IQR: 8–17), and 6 months (IQR: 5–7) for the three cohorts (Table 1).

**Table 1:** Baseline characteristics of training, testing, and prospective cohorts

Variable	Training cohort (*N* = 297)	Testing cohort (*N* = 104)	Prospective cohort (*N* = 30)	*P-*value
Follow up, months (median, IQR)	23 (16, 38)	11 (8, 17)	6 (5, 7)	<0.001
Gender				0.045
Female	89 (30%)	42 (40%)	14 (47%)	
Male	208 (70%)	62 (60%)	16 (53%)	
Age, years (median, IQR)	58 (49, 65)	58 (51, 65)	56 (42, 61)	0.269
<60	167 (56%)	59 (57%)	17 (57%)	0.996
≥60	130 (44%)	45 (43%)	13 (43%)	
CEA (U/ml, [median, IQR])	7 (3, 21)	7 (4, 21)	5 (3, 19)	0.359
CA199 (U/ml, [median, IQR])	12 (5, 59)	15 (5, 75)	9 (5, 37)	0.724
CA125 (U/ml, [median, IQR])	13 (9, 19)	12 (8, 19)	14 (10, 21)	0.596
Number of liver segments involved				0.018
≤2	169 (57%)	48 (46%)	14 (47%)	
3	56 (19%)	19 (18%)	3 (10%)	
4	37 (12%)	12 (12%)	5 (17%)	
≥5	35 (12%)	25 (24%)	8 (27%)	
Number of liver metastases				0.063
≤2	175 (59%)	53 (51%)	14 (47%)	
3–5	70 (24%)	23 (22%)	5 (17%)	
≥5	52 (18%)	28 (27%)	11 (37%)	
Maximum size of liver metastases exceeds 3 cm				0.004
No	148 (50%)	65 (63%)	23 (77%)	
Yes	149 (50%)	39 (38%)	7 (23%)	
Preoperative chemotherapy				0.010
No	142 (48%)	38 (37%)	7 (23%)	
Yes	155 (52%)	66 (63%)	23 (77%)	
Tumor site				<0.001
Left colon	244 (82%)	68 (65%)	26 (87%)	
Right colon	53 (18%)	36 (35%)	4 (13%)	
Pathological T stage				0.367
T0	6 (2.0%)	0 (0%)	1 (3.3%)	
T1	2 (0.7%)	0 (0%)	0 (0%)	
T2	27 (9.1%)	8 (7.7%)	3 (10%)	
T3	197 (66%)	73 (70%)	24 (80%)	
T4	65 (22%)	23 (22%)	2 (6.7%)	
Pathological N stage				0.235
N0	102 (34%)	43 (42%)	13 (43%)	
N1	146 (49%)	38 (37%)	13 (43%)	
N2	48 (16%)	22 (21%)	4 (13%)	
Pathological type				0.314
Infiltrating	45 (15%)	20 (19%)	3 (10%)	
Mass	89 (30%)	23 (22%)	6 (20%)	
Ulcerative	163 (55%)	61 (59%)	21 (70%)	
Differentiation				0.246
Highly	39 (13%)	9 (8.7%)	2 (6.7%)	
Moderately	215 (72%)	80 (77%)	27 (90%)	
Poorly	43 (14%)	15 (14%)	1 (3.3%)	
Intravascular tumor thrombus				0.313
No	204 (69%)	64 (62%)	22 (73%)	
Yes	93 (31%)	40 (38%)	8 (27%)	
Ki67	50 (30, 70)	60 (40, 70)	70 (40, 70)	0.016
HER2 stage^a^				0.555
0	213 (72%)	80 (78%)	22 (73%)	
1 +	49 (16%)	18 (17%)	6 (20%)	
2 +	23 (7.7%)	3 (2.9%)	2 (6.7%)	
3 +	12 (4.0%)	2 (1.9%)	0 (0%)	
Genes mutation				0.066
Wild type	145 (49%)	62 (62%)	17 (57%)	
Mutation^b^	152 (51%)	38 (38%)	13 (43%)	
BRAF mutation	23 (7.6%)	3 (2.9%)	2 (6.3%)	
EGFR mutation	1 (0.3%)	1 (1.0%)	0 (0%)	
KRAS mutation	71 (24%)	25 (24%)	11 (34%)	
NRAS mutation	28 (9.3%)	1 (1.0%)	0 (0%)	
PIK3CA mutation	34 (11%)	11 (11%)	2 (6.3%)	
UGT1A1 mutation	0 (0%)	1 (1.0%)	0 (0%)	

CA125, Cancer Antigen 125; CA199, Carbohydrate Antigen 19-9; CEA, Carcinoembryonic Antigen; HER2, human epidermal growth factor receptor 2; IQR, interquartile range.

^a^ 0 (Negative): No membrane positivity, 0% proportion; interpreted as negative;

1 + (Weakly Positive): Weak membrane positivity, ≤ 10% proportion; interpreted as negative;

2 + (Equivocal): Moderate to strong membrane positivity, 10–50% or ≥50% proportion; interpreted as equivocal, FISH testing recommended;

3 + (Positive): Strong membrane positivity, ≥ 50% proportion; interpreted as positive.

^b^ Eleven patients have double gene mutations.

Chi-squared tests were used to compare categorical variables, and the Mann–Whitney U-test was applied for continuous variables. A *P-*value < 0.05 was considered statistically significant.

The demographic and clinicopathological characteristics were generally similar across the three cohorts. The median age was comparable (approximately 56–58 years), and the proportion of male patients ranged from 53% to 70%. Serum biomarkers (CEA, CA199, CA125), the number of liver metastases, and pathological T and N stages showed no statistically significant differences (*P* > 0.05) (Table 1).

Pathological classification by binary criteria showed approximately one-third of patients classified as desmoplastic in all cohorts (33%, 37.5%, and 23%, respectively). The four-class classification revealed that the desmoplastic subtype predominated consistently across cohorts, with similar proportions of 75.1%, 72.1%, and 67% (Table 2). To further explore the associations between HGP types and clinical or pathological features, we visualized the distributions of binary and four-class pathological labels across relevant patient subgroups in the training cohort (Supplemental Digital Content Figure S1–S2, available at: http://links.lww.com/JS9/E586).

**Table 2:** HGP classifications in training testing and, prospective cohorts

Variable	Training cohort (*N* = 297)	Testing cohort (*N* = 104)	Prospective cohort (*N* = 30)	*P-*value
Binary pathological classification				0.337
Desmoplastic	98 (33%)	39 (37.5%)	7 (23%)	
Non-desmoplastic	199 (67%)	65 (62.5%)	23 (77%)	
Four-class pathological classification				0.175
Desmoplastic	223 (75.1%)	75 (72.1%)	20 (67%)	
Replacement	42 (14.1%)	12 (11.5%)	7 (23%)	
Pushing	21 (7.1%)	11 (10.6%)	0 (0%)	
Mixed	11 (3.7%)	6 (5.8%)	3 (10%)	

HGP, histopathological growth pattern. Chi-squared tests were used to compare categorical variables. A *P* value < 0.05 was considered statistically significant.

### Clinical characterization differences based on binary HGP classification

A univariate analysis was conducted on the clinical and pathological characteristics of binary HGP types (desmoplastic and non-desmoplastic) in 297 patients (Table 3). Among these, 98 were desmoplastic and 199 were non-desmoplastic. The analysis revealed no statistically significant differences in gender, age, number of liver segments involved, number of liver metastases, or maximum size of liver metastases (*P* > 0.05). No statistically significant differences were found in overall mutation rates or mutation rates for specific genes (e.g., KRAS, BRAF, NRAS) between the two groups.

**Table 3:** Clinicopathological characteristics of the training cohort based on binary HGP classification

Variable	Desmoplastic *N* = 98	Non-desmoplastic *N* = 199	*P-*value
Gender			0.239
Female	25 (26%)	64 (32%)	
Male	73 (74%)	135 (68%)	
Age, years (median, IQR)	58 (47, 64)	58 (49, 66)	0.361
<60	59 (60%)	108 (54%)	0.333
≥60	39 (40%)	91 (46%)	
CEA (U/ml, [median, IQR])	6 (3, 12)	9 (4, 29)	0.002
CA199 (U/ml, [median, IQR])	8 (4, 25)	18 (6, 90)	0.002
CA125 (U/ml, [median, IQR])	12 (9, 21)	13 (8, 19)	0.631
Number of liver segments involved			0.691
≤2	57 (58%)	112 (56%)	
3	21 (21%)	35 (18%)	
4	10 (10%)	27 (14%)	
≥5	10 (10%)	25 (13%)	
Number of liver metastases			0.639
≤2	61 (62%)	114 (57%)	
3–5	20 (20%)	50 (25%)	
≥5	17 (17%)	35 (18%)	
Maximum size of liver metastases exceeds 3 cm			0.651
No	47 (48%)	101 (51%)	
Yes	51 (52%)	98 (49%)	
Preoperative chemotherapy			0.833
No	46 (47%)	96 (48%)	
Yes	52 (53%)	103 (52%)	
Tumor site			0.036
Left colon	74 (76%)	170 (85%)	
Right colon	24 (24%)	29 (15%)	
Pathological T stage			0.319
T0	4 (4.1%)	2 (1.0%)	
T1	0 (0%)	2 (1.0%)	
T2	11 (11%)	16 (8.0%)	
T3	63 (64%)	134 (67%)	
T4	20 (20%)	45 (23%)	
Pathological N stage			0.061
N0	42 (43%)	60 (30%)	
N1	45 (46%)	101 (51%)	
N2	11 (11%)	37 (19%)	
Pathological type			0.229
Infiltrating	18 (18%)	27 (14%)	
Mass	33 (34%)	56 (28%)	
Ulcerative	47 (48%)	116 (58%)	
Differentiation			0.436
Highly	16 (16%)	23 (12%)	
Moderately	70 (71%)	145 (73%)	
Poorly	12 (12%)	31 (16%)	
Intravascular tumor thrombus			0.855
No	68 (69%)	136 (68%)	
Yes	30 (31%)	63 (32%)	
Ki67	50 (30, 70)	50 (30, 70)	0.613
HER2 stage^a^			0.604
0	71 (72%)	142 (71%)	
1 +	13 (13%)	36 (18%)	
2 +	9 (9.2%)	14 (7.0%)	
3 +	5 (5.1%)	7 (3.5%)	
Gene mutation			0.738
Wild type	51 (52%)	94 (47%)	
Mutation^b^	47 (48%)	105 (53%)	
BRAF mutation	9 (9.2%)	14 (6.9%)	
EGFR mutation	1 (1.0%)	0 (0%)	
KRAS mutation	20 (20%)	51 (25%)	
NRAS mutation	8 (8.2%)	20 (9.8%)	
PIK3CA mutation	9 (9.2%)	25 (12%)	
Median OS, months (95% CI)	53.6 (45.5-NA)	31.9 (27.8–45.1)	0.002
Median PFS, months (95% CI)	25.2 (18.10-38.3)	10.7 (8.07–13.6)	<0.001

CA125, Cancer Antigen 125; CA199, Carbohydrate Antigen 19-9; CEA, Carcinoembryonic Antigen; HER2, human epidermal growth factor receptor 2; HGP, histopathological growth pattern; IQR, interquartile range; OS, overall survival; PFS, progression-free survival.

^a^ 0 (Negative): No membrane positivity, 0% proportion; interpreted as negative;

1 + (Weakly Positive): Weak membrane positivity, ≤ 10% proportion; interpreted as negative;

2 + (Equivocal): Moderate to strong membrane positivity, 10–50% or ≥50% proportion; interpreted as equivocal, FISH testing recommended;

3 + (Positive): Strong membrane positivity, ≥ 50% proportion; interpreted as positive.

^b^ Five patients have double gene mutations.

Chi-squared tests were used to compare categorical variables, and the Mann–Whitney U-test was applied for continuous variables. The Log-rank test was used to compare the median survival between groups. A *P-*value < 0.05 was considered statistically significant.

A statistically significant difference was observed in the tumor site distribution between the two groups (*P* = 0.036). A higher proportion of desmoplastic patients had tumors in the right colon compared to non-desmoplastic patients (24% vs. 15%). Regarding TNM staging, desmoplastic patients had higher proportions of T0 and N0 stages compared to non-desmoplastic patients, though these differences were not statistically significant (*P-*values of 0.319 and 0.061, respectively). The levels of CEA (median 6 vs. 9) and CA199 (median 8 vs. 18) were significantly lower in desmoplastic patients compared to non-desmoplastic patients, with both differences being statistically significant (*P* = 0.002 and 0.002). These findings suggest that desmoplastic tumors may have distinct biological behaviors.

Overall survival (OS, average 53.6 months vs. 31.9 months, *P* = 0.002) and progression-free survival (PFS, average 25.2 months vs. 10.7 months, *P* < 0.001) were significantly longer in desmoplastic patients compared to non-desmoplastic patients.

### Clinical feature differences based on four-class HGP classification

Tumors were classified into four pathological growth patterns: desmoplastic, replacement, pushing, and mixed. We compared the clinicopathologic features, histologic, and molecular indices of patients across these four groups (Table 4). No significant differences were observed in age distribution, number of liver segments involved, number of metastases, maximum tumor diameter, preoperative chemotherapy, or primary tumor location among the groups. Additionally, the expression levels of Ki67, as well as the incidence of mutations in KRAS, NRAS, BRAF, and PIK3CA, were not significantly different.

**Table 4:** Clinicopathological characteristics of the training cohort based on four-class HGP classification

Variable	Desmoplastic *N* = 223	Replacement *N* = 42	Pushing *N* = 21	Mixed *N* = 11	*P-*value
Gender					0.183
Female	62 (28%)	15 (36%)	10 (48%)	2 (18%)	
Male	161 (72%)	27 (64%)	11 (52%)	9 (82%)	
Age, years (median, IQR)	58 (50, 66)	54 (47, 64)	61 (56, 66)	60 (44, 67)	0.366
<60	127 (57%)	26 (62%)	9 (43%)	5 (45%)	0.447
≥60	96 (43%)	16 (38%)	12 (57%)	6 (55%)	
CEA (U/ml, [median, IQR])	6 (3, 19)	12 (5, 38)	10 (4, 41)	18 (9, 98)	0.006
CA199 (U/ml, [median, IQR])	10 (5, 38)	40 (9, 147)	15 (5, 171)	38 (9, 255)	0.008
CA125 (U/ml, [median, IQR])	12 (9, 19)	14 (9, 19)	12 (9, 17)	17 (9, 24)	0.626
Number of liver segments involved					0.940
≤2	126 (57%)	23 (55%)	12 (57%)	8 (73%)	
3	43 (19%)	9 (21%)	3 (14%)	1 (9.1%)	
4	29 (13%)	5 (12%)	3 (14%)	0 (0%)	
≥5	25 (11%)	5 (12%)	3 (14%)	2 (18%)	
Number of liver metastases					0.848
≤2	132 (59%)	24 (57%)	13 (62%)	6 (55%)	
3–5	55 (25%)	8 (19%)	5 (24%)	2 (18%)	
≥5	36 (16%)	10 (24%)	3 (14%)	3 (27%)	
Maximum size of liver metastases exceeds 3 cm					0.629
No	107 (48%)	24 (57%)	12 (57%)	5 (45%)	
Yes	116 (52%)	18 (43%)	9 (43%)	6 (55%)	
Preoperative chemotherapy					0.624
No	108 (48%)	18 (43%)	12 (57%)	4 (36%)	
Yes	115 (52%)	24 (57%)	9 (43%)	7 (64%)	
Tumor site					0.428
Left colon	179 (80%)	38 (90%)	17 (81%)	10 (91%)	
Right colon	44 (20%)	4 (9.5%)	4 (19%)	1 (9.1%)	
Pathological T stage					0.990
T0	5 (2.2%)	1 (2.4%)	0 (0%)	0 (0%)	
T1	1 (0.4%)	1 (2.4%)	0 (0%)	0 (0%)	
T2	21 (9.4%)	3 (7.1%)	2 (9.5%)	1 (9.1%)	
T3	148 (66%)	27 (64%)	15 (71%)	7 (64%)	
T4	48 (22%)	10 (24%)	4 (19%)	3 (27%)	
Pathological N stage					0.258
N0	80 (36%)	11 (26%)	8 (38%)	3 (27%)	
N1	108 (49%)	23 (55%)	7 (33%)	8 (73%)	
N2	34 (15%)	8 (19%)	6 (29%)	0 (0%)	
Pathological type					0.124
Infiltrating	32 (14%)	5 (12%)	4 (19%)	4 (36%)	
Mass	75 (34%)	9 (21%)	3 (14%)	2 (18%)	
Ulcerative	116 (52%)	28 (67%)	14 (67%)	5 (45%)	
Differentiation					0.511
Highly	31 (14%)	5 (12%)	3 (14%)	0 (0%)	
Moderately	162 (73%)	30 (71%)	16 (76%)	7 (64%)	
Poorly	30 (13%)	7 (17%)	2 (9.5%)	4 (36%)	
Intravascular tumor thrombus					0.572
No	153 (69%)	27 (64%)	17 (81%)	7 (64%)	
Yes	70 (31%)	15 (36%)	4 (19%)	4 (36%)	
Ki67	50 (30, 70)	50 (30, 70)	40 (30, 70)	40 (20, 70)	0.730
HER2 stage^a^					0.019
0	161 (72%)	32 (76%)	15 (71%)	5 (45%)	
1 +	37 (17%)	7 (17%)	3 (14%)	2 (18%)	
2 +	16 (7.2%)	3 (7.1%)	3 (14%)	1 (9.1%)	
3 +	9 (4.0%)	0 (0%)	0 (0%)	3 (27%)	
Gene mutation					0.571
Wild type	106 (48%)	23 (55%)	12 (57%)	4 (36%)	
Mutation^b^	117 (52%)	19 (45%)	9 (43%)	7 (64%)	
BRAF mutation	17 (7.5%)	6 (14%)	0 (0%)	0 (0%)	
EGFR mutation	1 (0.4%)	0 (0%)	0 (0%)	0 (0%)	
KRAS mutation	54 (24%)	7 (16%)	6 (29%)	4 (36%)	
NRAS mutation	22 (9.7%)	4 (9.3%)	2 (9.5%)	0 (0%)	
PIK3CA mutation	27 (12%)	3 (7.0%)	1 (4.8%)	3 (27%)	
Median OS, months (95% CI)	51.0 (37.9–73.7)	26.4 (22.1–NA)	58.3 (28.3–NA)	20.0 (18.2-NA)	0.033
Median PFS, months (95% CI)	17.38 (14.72–20.9)	7.98 (5.48–12.2)	12.20 (5.15–34.2)	6.82 (5.21–NA)	<0.001

CA125, Cancer Antigen 125; CA199, Carbohydrate Antigen 19-9; CEA, Carcinoembryonic Antigen; HER2, human epidermal growth factor receptor 2; HGP, histopathological growth pattern; IQR, interquartile range; OS, overall survival; PFS, progression-free survival.

^a^ 0 (Negative): No membrane positivity, 0% proportion; interpreted as negative;

1 + (Weakly Positive): Weak membrane positivity, ≤ 10% proportion; interpreted as negative;

2 + (Equivocal): Moderate to strong membrane positivity, 10-50% or ≥50% proportion; interpreted as equivocal, FISH testing recommended;

3 + (Positive): Strong membrane positivity, ≥ 50% proportion; interpreted as positive.

^b^ Five patients have double gene mutations.

Chi-squared tests were used to compare categorical variables, and the Kruskal–Wallis H-test was applied for continuous variables. The Log-rank test was used to compare the median survival between groups. A *P-*value < 0.05 was considered statistically significant.

However, serum levels of CEA and CA199 were significantly higher in replacement and mixed-type patients compared to the other groups. Significant differences were also found in HER2 status across the groups, with the mixed-type group having a lower proportion of HER2-negative patients (45%) and a higher proportion of HER2-strong positive patients (27%) compared to the others.

Survival analysis revealed statistically significant differences in OS and PFS among the groups. Patients with pushing-type tumors had the longest OS (58.3 months), while those with desmoplastic tumors had the longest PFS (17.38 months). In contrast, mixed-type patients had the shortest OS (20.0 months) and PFS (6.82 months), and replacement-type patients had relatively shorter OS (26.4 months) and PFS (7.98 months).

### Predictive performance of binary HGP classification

**Figure 3.**
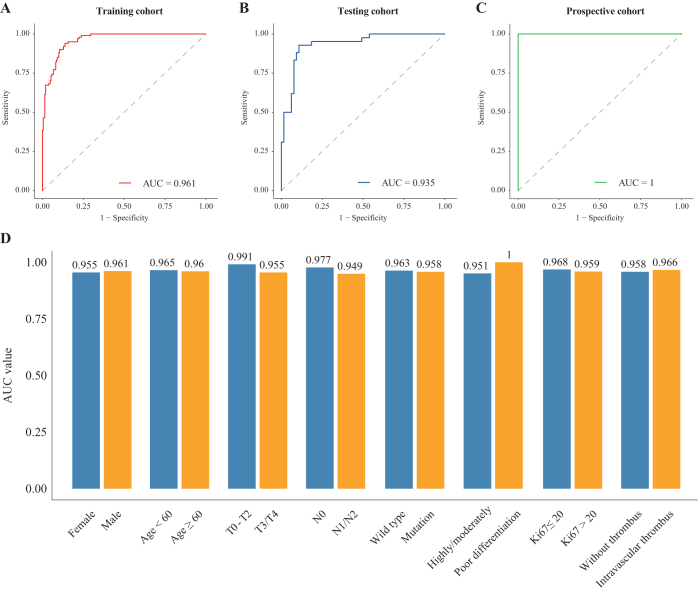
Binary HGP classification prediction performance. Performance in (A) the training cohort, (B) the testing cohort, and (C) the prospective cohort; (D) subgroup analysis of AUC values. HGP, histopathological growth pattern.

The performance of the COFFEE binary HGP classification model was evaluated across multiple cohorts and subgroups, demonstrating strong predictive capabilities. In the training cohort, the model achieved an AUC of 0.961 (Fig. 3A), in the testing cohort, 0.935 (Fig. 3B), and in the prospective cohort, a perfect AUC of 1.000 (Fig. 3C), indicating both robustness and high generalizability to future clinical data.

Subgroup analysis in the training cohort (Fig. 3D) revealed consistent AUC values across various clinical and pathological features. The highest AUCs were observed in the T0–T2 stage subgroup (AUC = 0.991) and the poorly differentiated tumor subgroup (AUC = 1). Other key subgroups also showed high AUCs: gender (female: 0.955, male: 0.961), age (age <60 years: 0.965, age ≥60 years: 0.960), lymph node status (N0: 0.977, N1/N2: 0.949), and Ki67 status (Ki67 ≤ 20: 0.968, Ki67 > 20: 0.959) (Supplemental Digital Content Table S1, available at: http://links.lww.com/JS9/E586). These results confirm that the COFFEE model delivers reliable predictions across a wide range of clinical and pathological contexts.

### Evaluating the performance of four-class HGP classification

**Figure 4.**
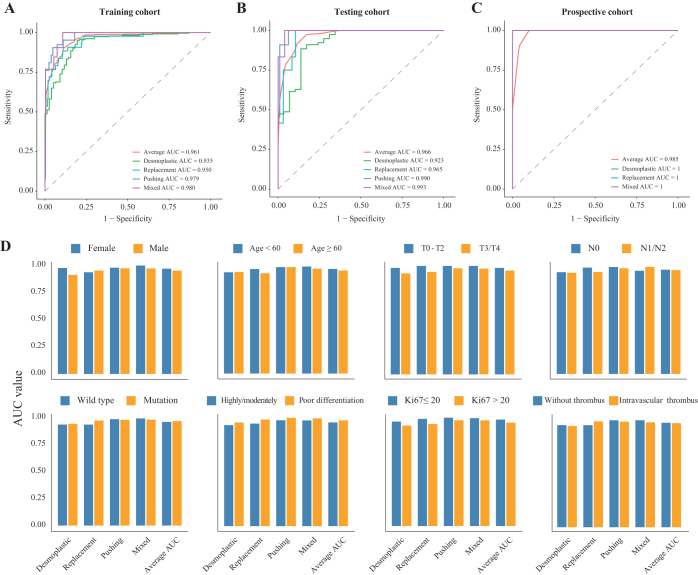
Four-class HGP classification prediction performance. Performance in (A) the training cohort, (B) the testing cohort, and (C) the prospective cohort; (D) subgroup analysis of AUC values. HGP, histopathological growth pattern.

The COFFEE four-class HGP classification model was evaluated across multiple cohorts, demonstrating strong performance and generalizability. In the training cohort, the model achieved an average AUC of 0.961, with the pushing and mixed subtypes showing the highest AUCs of 0.979 and 0.980, respectively, while the desmoplastic and replacement subtypes had slightly lower AUCs of 0.935 and 0.950 (Fig. 4A). In the testing cohort, the average AUC was 0.966, with pushing and mixed subtypes again performing well (AUCs of 0.990 and 0.993) and desmoplastic and replacement subtypes achieving AUCs of 0.923 and 0.965 (Fig. 4B). The prospective cohort exhibited excellent generalizability, achieving an average AUC of 0.985, with all subtypes reaching perfect AUCs of 1.0 (Fig. 4C).

Subgroup Analysis in the training cohort highlighted robust performance across various clinical and pathological categories (Fig. 4D). The model achieved consistently high AUCs, with female and male patients reaching 0.971 and 0.953, respectively. The T0–T2 stage showed perfect classification (AUC = 1) for the pushing, mixed, and replacement subtypes, while the T3/T4 stage had slightly lower performance. Similarly, lymph node status (N0: 0.962, N1/N2: 0.958) and Ki67 status (Ki67 ≤ 20: 0.982, Ki67 > 20: 0.954) indicated strong performance across all classes. Tumors with poor differentiation achieved an AUC of 0.976, while highly/moderately differentiated tumors had an AUC of 0.957 (Supplemental Digital Content Table S1, available at: http://links.lww.com/JS9/E586). The COFFEE model demonstrated robust diagnostic performance across cohorts and subgroups, with slight reductions in accuracy noted in specific categories such as intravascular tumor thrombus and T3/T4 stages.

### Effect of AI-assisted diagnostic performance in a prospective cohort

We evaluated the diagnostic performance of an AI-assisted model compared to pathologists using a prospective validation dataset. The analysis included 30 cases reviewed by three groups of pathologists with varying experience levels, junior (*n* = 3), mid-level (*n* = 3), and senior pathologists (*n* = 3), to assess diagnostic accuracy and speed.

**Figure 5.**
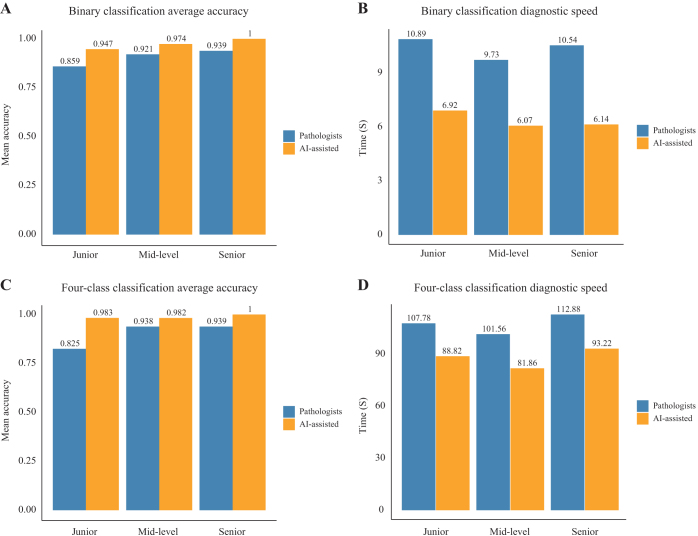
Impact of AI-assisted diagnostic performance in the prospective cohort. (A) Binary HGP classification diagnostic accuracy and (B) diagnostic speed; (C) Four-class HGP classification diagnostic accuracy, and (D) diagnostic speed. HGP, histopathological growth pattern.

For binary HGP classification, the diagnostic accuracy was 85.9%, 92.1%, and 93.9% for junior, mid-level, and senior pathologists, respectively. In comparison, the AI-assisted model achieved accuracies of 94.7% (junior), 97.4% (mid-level), and 100% (senior) (Fig. 5A). Regarding diagnostic speed, junior pathologists required 10.89 s per case, mid-level pathologists 9.73 s, and senior pathologists 10.54, while the AI-assisted model reduced this time to 6.92 s (junior), 6.07 s (mid-level), and 6.14 s (senior) per case (Fig. 5B). Detailed performance metrics, including accuracy, sensitivity, specificity, PPV, NPV, and AUC, are presented in Supplemental Digital Content Table S2, available at: http://links.lww.com/JS9/E586 for COFFEE alone, pathologists, and AI-assisted pathologists.

For four-class HGP classification, the diagnostic accuracy was 82.5%, 93.8%, and 93.9% for junior, mid-level, and senior pathologists, respectively, while the AI-assisted model achieved 98.3% (junior), 98.2% (mid-level), and 100% (senior) (Fig. 5C). Diagnostic speed showed similar trends, with junior pathologists taking 107.78 s per case, mid-level pathologists 101.56 s, and senior pathologists 112.88 s. The AI-assisted model reduced this to 88.82 min (junior), 81.86 min (mid-level), and 93.22 min (senior) per case (Fig. 5D). Additional performance metrics for this classification task are summarized in Supplemental Digital Content Table S3, available at: http://links.lww.com/JS9/E586.

These findings demonstrate that the AI-assisted diagnostic model consistently outperformed pathologists in accuracy and significantly reduced diagnostic time across all experience levels.

### Visualization and interpretation

In this study, we focused on the image regions and significant features influencing the output of the pathology-based deep learning model. This effort aimed to enhance clinicians’ understanding of the network’s predictions and provide insights into tumor zones.

**Figure 6.**
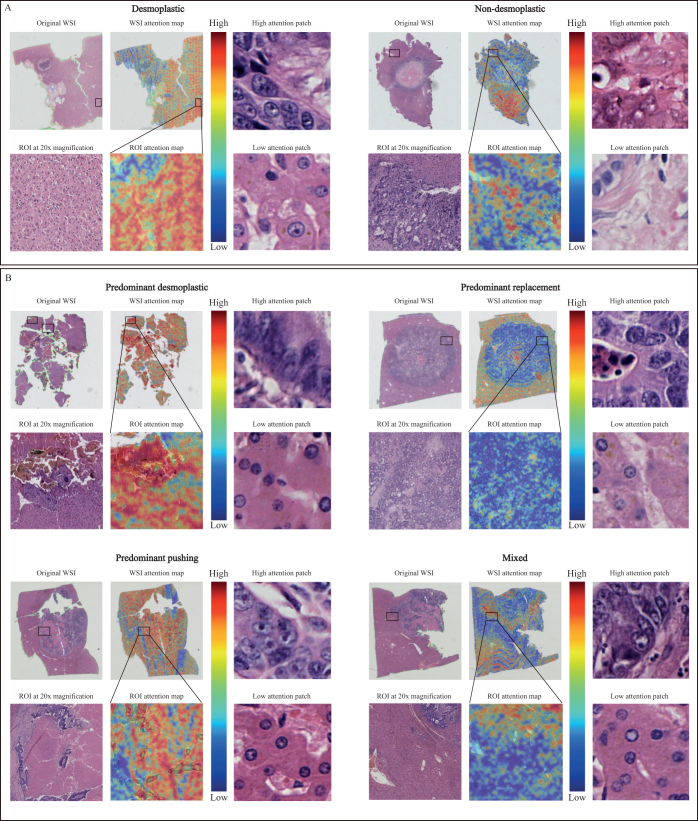
Histopathological analysis and attention mapping of binary HGP classification and four-class HGP classification WSI in CRLM. (A) Attention mapping of binary HGP classification; (B) Attention mapping of four-class HGP classification. HGP, histopathological growth pattern; CRLM, colorectal liver metastasis.

The heatmaps generated from our analysis clearly illustrate how the model’s attention mechanism functions within CRLM pathology (Fig. 6A and B). Warm colors (such as red) indicate regions with a decisive impact on the model’s prediction of the pathological subtype, while cooler colors (such as blue) suggest minimal influence on the model’s outcome. Moreover, darker shades represent stronger network responses, with higher attention weights, highlighting the model’s increased focus on these specific regions. In the binary HGP classification heatmap, the red-dominated areas primarily captured high-level semantic features of the tumor extracted from WSI patches, which were crucial in classifying the WSI as desmoplastic. In contrast, the blue-dominated areas reflected normal WSI structural features, such as well-defined boundaries, intact tissue shape, and texture, which led to the classification of the WSI as non-desmoplastic (Fig. 6A). In the four-class HGP classification heatmaps, each WSI subtype exhibited distinctive features, and the attention mechanism of the COFFEE model effectively identified high-risk and low-risk regions within the tissue, mirroring the characteristics of the four subtypes in the WSI (Fig. 6B).

These results demonstrate that the visualizations in this study provide valuable insights for pathologists and clinicians in the pathological classification of colorectal cancer liver metastasis. They enhance the interpretability of deep learning models in pathology predictions, enabling precise localization of high-risk areas, which significantly improves diagnostic accuracy and treatment planning.

## Discussion

HGP is crucial for patients with CRLM, especially for post-surgery therapeutic strategy, including chemotherapy and target therapy, etc. However, an AI pathological predictive model focus on HGP classification has not been developed before. This study is the first to develop an pathological AI model for prenedicting HGP classifications, and to validate its performance through a prospective randomized pilot study. In this study, we develop the first HGP classification AI model COFFEE in global using the DINO and TransMIL method, aimed at predicting the type of HGPs following complete surgical treatment of CRLM. Both the retrospective and prospective show consistent results that both the two-class and four-class HGP predictive AI models COFFEE accurately predicted the classification of HGP, with the four-class model could further guide treatment. The desmoplastic phenotype was independently associated with better OS and DFS outcomes. Particularly noteworthy, in the prospective cohort, the AI-assisted pathologists surpassed cytopathologists alone, exhibiting significantly higher AUC, specificity, and accuracy. Thus, COFFEE holds promise as an additional tool for accurate and efficient HGP classification and screening potential eligible patients for clinical trials involving HGP classification.

Based on the clinical trials’ results of CRLM, such as TRICE and CAIRO5, it is evident that patients with initially unresectable colorectal cancer liver metastases can achieve significant OS benefits if they can be converted to a resectable state^[22,23]^. Patients should be categorized by tumor location and genetic phenotype: left-sided wild-type tumors and right-sided and/or mutant-type tumors. For left-sided wild-type tumors, the preferred treatment is dual-agent chemotherapy combined with EGFR monoclonal antibodies. For right-sided and/or mutant-type tumors, triple-agent chemotherapy with bevacizumab is the first choice, provided the patient can tolerate it. If triple-agent chemotherapy is not tolerable, dual-agent chemotherapy with bevacizumab can be considered as an alternative. HGP significantly influence patient prognosis and response to chemotherapy and targeted therapies. dHGP typically associated with enhanced vessel growth which shows better responses to oxaliplatin-based adjuvant chemotherapy and anti-angiogenic agents such as bevacizumab. In contrast, rHGP, characterized by vessel co-option, which is less responsive to anti-angiogenic therapy but can benefit from chemotherapy regimes with triplet or dual modalities combined with targeted agents—consistent with CAIRO-5 and TRICE trial subgroup analyses. pHGP, associated with mixed responses, shows favorable outcomes when treated with combination chemo plus EGFR inhibitors, especially in RAS wild-type tumors. Previous studies have shown that dHGP is associated with longer OS and PFS in CRLM, while non-dHGP predicts poorer outcomes^[7,24–27]^. The four-class HGP classification also predicts treatment response. dHGP responds better to liver-limited recurrence, while replacement rHGP and pushing pHGP are linked to multi-organ recurrence^[28–30]^. Additionally, chemotherapy can alter HGP classification, with dHGP and pHGP responding more favorably to specific therapies, whereas rHGP shows poor response^[31]^. Consistent with previous studies, both the binary classification model and four-Class classification model demostrated that dHGP is associated with longer OS and PFS in patients in patients with CRLM. Moreover, promising outcomes were observed for the four-class classification model, which exhibited an exceptionally high AUC of 0.961. The COFFEE demonstrated high sensitivity and specificity, indicating its capability to differentiate dHGP and from rHGP and pHGP. Therefore, COFFEE can potentially lead to earlier diagnosis for patients with CRLM, thereby guiding the personalized management and enhancing patient outcomes.

With the rapidly development of artificial intellgence, apply deep learning on WSIs to help boost the efficiency of the pathologists’ diagnosis is very promising. An increasing number of pathology laboratories are digitising glass slides into high-resolution WSIs. This creates an opportunity to develop algorithms based on machine learning and artificial intelligence that can extract clinically useful information from, for example, WSIs of H&E-stained tumour sections, implemented this approach to score the HGPs of liver metastases in an automated way^[32–34]^. Jeroen Van der Laak’s team developed an algorithm to distinguish CRLM with 100% desmoplastic HGP from those with any non-desmoplastic HGP. The algorithm compresses WSIs using an encoder, reducing dimensionality and noise. A convolutional neural network then classifies the images, achieving an AUC of 0.895 in predicting HGP. The algorithm also stratified 337 patients into two risk groups predicting overall survival (HR: 2.35, *P* < 0.001), showing the prognostic potential of this approach in assessing liver metastases. However, an AI pathological predictive AI model focus on HGP four classification has not been developed yet. This study develop a pathological AI model for four HGP classifications and to validate its performance through a prospective, comparative reader study involving parallel independent pathologist groups. In the retrospective evaluation, the proposed AI model achieved an AUC of 0.961. Consistent precision diagnostic performance was observed in the prospective evaluation, exhibiting significantly higher values in AUC, specificity, accuracy, and sensitivity. Particularly noteworthy, the diagnostic performance for dHGP, non HGP, rHGP, and pHGP, which hold the promise for the personalized post-surgery treatment, as dHGP and pHGP are more sensitive to a triplet chemotherapy regimen plus cetuximab and bevacizumab, respectively, while rHGP shows a poor response to both treatments^[31]^. Thus, COFFEE shows the potential as an adjunct tool not only for HGP classificaion, but also guiding the therapeutic strategy.

In this study, we developed the AI-based HGP predictive model COFFEE using retrospective data and validated its high diagnostic performance in both retrospective and prospective settings. Notably, in a prospective comparative reader study, the two-class COFFEE model significantly improved diagnostic accuracy, AUC, and specificity compared to unaided pathologists, particularly benefiting junior readers. The four-class model also demonstrated strong potential as an assistive tool for comprehensive HGP subtyping. These findings suggest that COFFEE can guide treatment decisions and potentially improve patient outcomes.

To facilitate clinical integration, COFFEE could be embedded into digital pathology workflows, providing real-time support for HGP assessment and reducing interobserver variability. Its use may be particularly beneficial in non-specialist settings or high-throughput clinical environments. Additionally, COFFEE’s ability to distinguish dHGP from rHGP and pHGP, with AUCs of 0.935, 0.950, and 0.979, respectively, could support personalized treatment planning and eligibility screening for clinical trials that stratify patients by HGP type.

Looking forward, we aim to build a more comprehensive AI system by incorporating multi-omics data, including gene expression, treatment history, immunohistochemical markers, and radiomics features. This would allow COFFEE not only to classify HGPs more precisely but also to predict tumor behavior, treatment response, and long-term prognosis. Recent advances in generative AI, such as the inClinico platform, highlight the potential of multimodal models to predict clinical trial outcomes and guide drug development^[35,36]^.

In summary, this study demonstrates the feasibility and clinical utility of COFFEE in classifying HGPs in CRLM patients with high accuracy. By outlining a pathway for clinical deployment and future multi-omics integration, we present COFFEE as a scalable and impactful tool for advancing precision oncology.

## Limitation

Nevertheless, this study has several limitations. First, this study employed a single-center design, which inherently restricts the generalizability of the results, therefore, future validation studies involving larger, multi-center cohorts are essential to evaluate the robustness and external validity of the COFFEE model. Second, postoperative treatment regimens were not collected or analyzed in this study, which limits the assessment of potential associations between various HGP types and therapeutic responses, thereby restricting the clinical utility of the findings. Subsequent studies should incorporate comprehensive postoperative treatment information to better understand these relationships. Third, there are inherent potential limitations in the study’s design, such as selection bias due to retrospective patient selection criteria and the exclusion of biopsy specimens and treated lesions. These factors may have influenced patient representativeness and potentially biased the model’s performance. Further prospective studies designed to minimize selection bias and include diverse patient populations are necessary. Lastly, the COFFEE model has inherent limitations. While demonstrating high accuracy, its performance may vary across different subgroups not represented in this study’s datasets. Additionally, the model’s interpretability, while enhanced through visualization techniques, remains limited and could benefit from further refinement to ensure transparency and usability in clinical decision-making. Future developments should focus on enhancing model explainability and robustness across diverse clinical contexts.

## Conclusions

This study presents the first AI-based pathological model for predicting HGP in colorectal cancer liver metastasis CRLM. The COFFEE demonstrated high accuracy in both binary classification and four-class classification HGP classification, with the four-class model offering additional insights for guiding post-surgery treatment strategies. Furthermore, in a prospective randomized trial, AI-assisted pathologists outperformed traditional methods, highlighting the COFFEE’s potential for improving diagnostic precision and clinical decision-making.

## Data Availability

Data are available with publication from the corresponding author upon reasonable request.

## Research registration unique identifying number (UIN)

This study was registered at ClinicalTrials.gov (NCT06936098): https://register.clinicaltrials.gov/prs/app/template/ViewPastRelease.vm?version=67&popup=true&uid=U0004NAT&ts=26&sid=S000F84V&cx=pob4yf.
